# Association between carbohydrate-to-fiber ratio and endometriosis in U.S. women: mediating role of neutrophil-percentage-to-albumin ratio

**DOI:** 10.1080/07853890.2026.2725512

**Published:** 2026-09-19

**Authors:** Yanli Hu, Chunmei Xiao, Qiao Zheng, Yuhan Zhang, Qian Ran, Yingying Zhang, Zhengchun Yang, Suzhen Ran

**Affiliations:** a Department of Ultrasonography, Chongqing Health Center for Women and Children, Chongqing, China; b Department of Ultrasonography, Women and Children’s Hospital of Chongqing Medical University, Chongqing, China

**Keywords:** endometriosis, carbohydrate-to-fiber ratio, neutrophil percentage-to-albumin ratio, NHANES, cross-sectional study

## Abstract

**Objectives:**

Associations between carbohydrate and fiber intake and endometriosis (EM) have been inconsistent, and no study has evaluated the combined effect of the carbohydrate-to-fiber ratio (CFR) or its inflammatory mechanisms.

**Materials and Methods:**

TThis cross-sectional study used data from two NHANES cycles (2003-2004 and 2005-2006), including 2,400 women aged 20-54 years. Weighted multivariable logistic regression assessed associations between CFR and physician-diagnosed EM, with restricted cubic splines used to identify non-linear relationships. The neutrophil percentage-to-albumin ratio (NPAR) was examined as a potential inflammatory mediator.

**Results:**

CFR was positively associated with EM in all models (fully adjusted OR: 1.02, 95% CI: 1.01-1.04). A non-linear relationship emerged, with a turning point at CFR 17.3 (p for nonlinearity = 0.037): below this threshold, a one-unit increase in CFR raised EM odds by 11% (OR: 1.11, 95% CI: 1.00-1.22), while no significant association was observed above it (OR: 1.02, 95% CI: 1.00-1.04). The fully adjusted OR for the highest CFR quartile (≥23.1) was 2.17 (95% CI: 1.06-4.14). NPAR partially mediated the CFR-EM association (proportion mediated: 5.16%). Subgroup analyses showed a consistent positive association, with a significant interaction only for hypertension status (*p* for interaction = 0.040).

**Conclusions:**

This study identifies a novel association between CFR and EM prevalence, partially mediated by NPAR. It is the first to apply CFR as a specific dietary measure, offering a clinically actionable tool for dietary assessment and patient counseling. Given the cross-sectional design, causal inference cannot be drawn, and prospective studies are needed to confirm these findings.

## KEY MESSAGES

CFR may be a useful dietary metric associated with EM prevalence, with a specific threshold of 17 for risk stratification.

CFR offers a simple, actionable metric for dietary counseling that emphasizes reducing refined carbohydrates and increasing fiber intake.

## 1. Introduction

Endometriosis (EM) is a highly prevalent disease that affects a large percentage of the female population. Recent studies have estimated that as much as 10% of women of reproductive age have been diagnosed with EM [1,2]. EM is a chronic inflammatory disease that involves the atypical growth of endometrial tissue outside of the uterus. Furthermore, EM also poses a large economic burden on society as a whole, with recent studies suggesting that the total cost of the disease in the United States alone was $69.4 billion, when accounting for healthcare costs, lost of productivity, and other indirect costs [3,4]. The majority of clinical research has focused on the link between EM and estrogen, since endometriotic tissues are known to be hormone-dependent. However, EM has also been associated with the state of chronic systemic inflammation, the dysregulation of immune system responses, and increased risk of metabolic diseases [5,6]. This relationship has been considered to be the result of increased levels of pro-inflammatory cytokines, oxidative stress, and overall dysregulated immune system activity, which supports the growth of endometriotic tissues outside of the uterus [7,8]. It has also been suggested that nutritional factors can influence the development of EM due to their effects on systemic inflammation, hormonal balance, and metabolic health, which are all underlying factors that contribute to the inflammatory-metabolic profile of EM [9,10].

Dietary carbohydrates and fiber are two macronutrients that have opposing functions within the human body. Carbohydrates are the main source of energy for many of the cells that make up the body. However, the consumption of carbohydrates in excess, or highly processed forms, has been shown to be associated with several negative health outcomes, including rapid spikes in blood sugar levels, insulin resistance, and inflammation [11,12]. On the other hand, dietary fiber has been shown to have multiple anti-inflammatory effects. Fiber refers to non-digestible carbohydrates, such as cellulose, hemicellulose, and pectin, which can modulate the composition of the gut microbiota and short-chain fatty acid production, as well as lower postprandial blood glucose and insulin levels [13,14]. The carbohydrate-to-fiber ratio (CFR) refers to the ratio of the total amount of carbohydrates in a diet, when compared to the total amount of fiber. A low CFR can be an indicator of higher quality diet that is higher in fiber and lower in refined carbohydrates, while a high CFR can indicate a lower quality diet that is higher in refined carbohydrates and lower in fiber [15]. CFR can be a better indicator of diet quality, when compared to simply looking at the total carbohydrate or fiber intake, since this takes into account the overall balance of the two types of nutrients within a diet. In addition, the CFR can provide information on the overall dietary pattern, with low CFR indicating a higher quality dietary pattern that is high in whole grains, fruits, and vegetables [16]. Research has shown that higher CFR is associated with increased inflammation, insulin resistance, oxidative stress, and metabolic syndrome, which are all factors that are implicated in EM [17,18].

Evidence on dietary carbohydrate and fiber intake, and EM risk remains limited and inconsistent. Case-control studies have suggested the protective effects of fiber *via* reduced estrogen and inflammation [19,20], while prospective cohorts have reported mixed or null results [21,22]. Furthermore, carbohydrate studies vary due to the differences in quality assessment and confounders [23,24]. These inconsistencies may have stemmed from the small samples, the recall bias, and ignoring the combined carbohydrate and fiber effects. No study has examined the CFR or its inflammatory mechanisms in EM.

The neutrophil percentage-to-albumin ratio (NPAR) is a novel marker of systemic inflammation and nutrition, outperforming traditional indices. This combines the neutrophil percentage (immune activation) and serum albumin (inversely linked to inflammation) [25,26]. NPAR can predict the outcomes in chronic inflammatory diseases [27,28], and is relevant to EM, as characterized by neutrophil activation and hypoalbuminemia [29]. The investigators hypothesized that higher CFR is positively associated with EM prevalence, potentially through systemic inflammation reflected by NPAR, thereby linking diet quality to reproductive health.

The present study bridges the gap in existing literature by addressing two knowledge gaps using the nationally representative National Health and Nutrition Examination Survey (NHANES) data: the association between CFR and EM prevalence, and the mediating role of NPAR in this relationship. The present study aimed to (1) assess the association between higher CFR and EM prevalence among U.S. women, and (2) determine the extent to which NPAR mediates the association between CFR and EM, which might elucidate the inflammatory mechanisms that link diet quality with EM. To our knowledge, the present population-based investigation is the first to examine CFR and EM with an inflammation-based mediation analysis. The results might provide novel evidence to support dietary guidance for EM prevention, and improve the understanding of the nutritional-inflammatory mechanisms that affect reproductive health.

## 2. Materials and methods

### 2.1. Study design and participants

**Figure 1.**
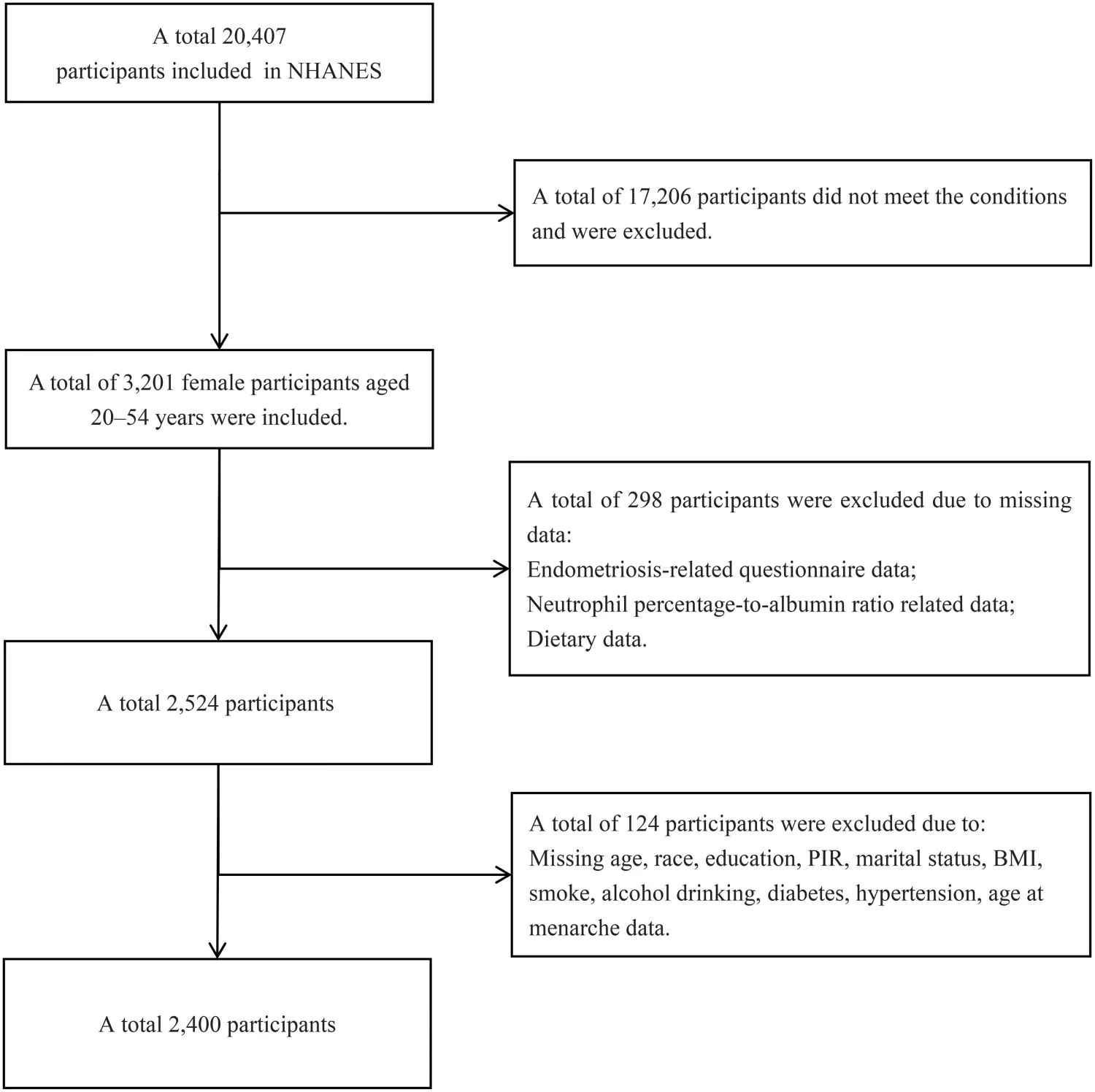
Flowchart for the participant selection. NHANES, National Health and Nutrition Examination Survey.

NHANES is a nationally representative health and nutrition public database released by the Centers for Disease Control and Prevention (CDC), and data is released every two years. The present study included two consecutive survey cycles (2003–2004 and 2005–2006) from NHANES. These two cycles were selected because these were the only NHANES cycles in which the self-reported endometriosis questionnaire (administered from 1999 to 2006) and the dietary and dietary-supplement carbohydrate and fiber data required to derive the CFR (available from 2003 onwards) were concurrently collected. Furthermore, the overlap defined the study period. No a priori sample-size calculation was performed, since this was a secondary analysis that included all eligible participants. Female participants within 20–54 years old were included, while female participants with missing EM questionnaires, missing food and supplement intakes, or missing NPAR were excluded. Furthermore, participants with missing covariates were excluded. A total of 2,400 female participants were included in the analysis. The detailed participant-selection process is presented in Figure 1.

### 2.2. Variables

#### 2.2.1. Definitions of EM

EM was defined through a questionnaire: ‘Have you ever been told by a doctor or other health professionals that you have endometriosis?’ (reference period: interview date, participants within 20–54 years old). Participants who reported ‘yes’ were defined as cases, while participants who reported ‘no’ were defined as controls [20].

#### 2.2.2. Definition of CFR

The main variable of interest was the CFR. CFR was calculated as the ratio of the 2-day average total carbohydrate intake to the 2-day average total fiber intake, with each nutrient summed from both food and dietary supplements across the two 24-hour dietary recalls. Quartile cut-offs were derived from the CFR distribution of the included study population (*n* = 2,400). Participants were categorized as Q1 (<13.2), Q2 (13.2–17.4), Q3 (17.4–23.1), and Q4 (≥23.1) [30].

#### 2.2.3. Definition of NPAR

The white blood cell count was measured using an automated hematology analyzer (Coulter^®^ DxH 800), and the neutrophil percentage was calculated using the Coulter VCS system. NPAR was defined, as follows: neutrophil percentage (of the total white blood cell count) (%) × 100/serum albumin (g/dL) [31]. NPAR was individually computed for each participant before its distribution was summarized within groups. Since NPAR is a nonlinear ratio, the group-level mean NPAR represented the average of individual NPAR values, and could not be reproduced from the group means of neutrophil percentage, white-blood-cell count, and albumin (*i.e.* E[X/Y] ≠ E[X]/E[Y]).

#### 2.2.4. Other covariates

The other covariates included age, race, poverty income ratio (PIR), education level, marital status, body mass index (BMI), smoking, alcohol consumption, hypertension, diabetes, and age at menarche. Age was measured at the screening phase. Race was categorized, as follows: Mexican American, non-Hispanic black, non-Hispanic white, other Hispanic, and other races. Education was classified, as follows: <high school diploma, high school diploma/equivalent, and > high school diploma. Marital status was categorized, as follows: widow/divorced/separated, married/cohabiting, and unmarried. PIR was divided into three groups: PIR ≤1.30, 1.30–3.50, and PIR >3.50 [32].

Smoking was categorized by asking the participants the following: ‘Have you smoked at least 100 cigarettes in your life?’ Participants who answered ‘no’ to the question were categorized as ‘never smokers’. Participants who answered ‘yes’ to the question were further asked the following: ‘Do you currently smoke?’ If the answer was ‘yes’, the participant was categorized as ‘current smoker’, while if the answer was ‘no’, the participant was categorized as ‘former smoker’ [33]. Alcohol consumption was defined as self-reported drinking ‘at least 12 drinks per year’ or ‘drinking alcohol’. If the information was missing, alcohol consumption was categorized as ‘unknown’ [34]. Hypertension was defined as systolic blood pressure ≥130 mm Hg, diastolic blood pressure ≥80 mm Hg, or current use of antihypertensive medication, or with a prior diagnosis of hypertension [35]. Diabetes was defined as receiving treatment for hyperglycemia, which is a medical diagnosis of diabetes, hemoglobin A1c ≥6.5%, fasting glucose ≥126 mg/dL, or 2-hour glucose ≥200 mg/dL [36].

### 2.3. Statistical analysis

NHANES involved a complex, multistage probability sampling design for selecting nationally representative participants. For all analyses, the sample weights, clustering, and stratification were accounted for, in order to produce national representative estimates. The baseline characteristics of all participants were described according to EM status. Normally distributed continuous variables were presented in mean ± standard deviation (SD). For non-normally distributed variables, the median with interquartile range (M Q1, Q3]) was reported. Comparisons between groups were conducted using *t*-test or Kruskal-Wallis H test. Categorical variables were presented in count and percentage (*n* %]), and compared using *χ^2^* or Fisher’s exact test. Multivariable logistic regression models with the survey package in R were used to evaluate the association between CFR and EM. Three models were fitted: an unadjusted model (crude), a model adjusted for age, race, education, PIR, and marital status (model I), and a fully adjusted model (model II), which was additionally adjusted for BMI, smoking, alcohol consumption, diabetes, hypertension, and age at menarche. Restricted cubic splines were used to test the nonlinear associations between CFR and EM. If a nonlinear relationship was detected, the two-segment linear regression model was used to explore the threshold effect of CFR on EM. Subgroup analysis was performed to explore the potential heterogeneity and interactions of different populations. Furthermore, mediation analysis was conducted to determine whether NPAR mediated the CFR-EM association. Using the R mediation package, the indirect, direct, and total effects of CFR on EM mediated by NPAR were estimated. The mediation proportion was calculated, as follows: indirect effect/total effect. The indirect, direct, and total effects, together with their 95% confidence intervals (CIs), were estimated using 1000 bootstrap resamples, and the mediation proportion was reported as a point estimate. The primary analysis was based on complete cases. Multiple imputation was applied only as a sensitivity analysis, and because the two approaches produced closely comparable estimates (Supplementary Table 5). Complete-case results were reported as the main findings. Three sensitivity analyses were performed. First, the logistic regression analysis was repeated without imputation. Second, dietary protein, energy intake, and physical activity were included in model II, in order to adjust for the influence of overall diet quality and lifestyle factors. Third, the R mice package was used to replace the missing covariates with multiple imputation. Then, the association between CFR and EM was reassessed. All statistical analyses were performed using R (V4.4.1). A two-sided *p* < 0.05 was considered statistically significant.

## 3. Results

### 3.1. Baseline characteristics

The baseline characteristics of the 2,400 participants are summarized in Table 1, according to EM status. Compared with the no EM group, women in the EM group were significantly older (42.00 *vs.* 37.00 years old, *p* < 0.001), and had a higher percentage of non-Hispanic white participants (84.97% *vs.* 67.77%, *p* < 0.001). In addition, women with EM had a higher level of education (>high school diploma, 68.17% *vs.* 63.28%) and a widow/divorced/separated status (24.69% *vs.* 14.62%). Notably, although there was no statistical difference, the EM group had slightly lower BMI (25.45 *vs.* 26.26 kg/m^2^), current smokers (29.51% *vs.* 25.26%), and alcohol consumption (73.82% *vs.* 68.02%). Furthermore, the EM group had higher CFR (17.77 *vs.* 17.07, *p* = 0.067), and lower fiber intake (12.00 *vs.* 12.70 gm), despite the similar carbohydrate intake. Moreover, the EM group exhibited a trend towards a higher NPAR (14.85 *vs.* 14.17, *p* = 0.072), suggesting a potential difference in inflammatory status. There was no significant difference in the prevalence of diabetes, hypertension status, or age at menarche between the two groups. The flowchart for the screening process of the participants is presented in Figure 1.

**Table 1.** Characteristics of NHANES participants.

Characteristic	Total (*N* = 2,400)	Without EM (*n* = 2,229)	With EM (*n* = 171)	*p*-value
Age	38 (29, 46)	37 (29, 46)	42 (37, 47)	<0.001
**Race**				<0.001
Mexican American	508 (9.1%)	494 (9.8%)	14 (2.4%)	
Other Hispanic	90 (3.5%)	86 (3.7%)	4 (1.2%)	
Non-Hispanic white	1,151 (69.3%)	1,041 (67.8%)	110 (85.0%)	
Non-Hispanic black	536 (13.1%)	499 (13.5%)	37 (9.1%)	
Other race	115 (5.0%)	109 (5.3%)	6 (2.4%)	
**Education**				0.248
< High school diploma	504 (13.8%)	486 (14.4%)	18 (8.2%)	
High school diploma/equivalent	515 (22.5%)	478 (22.4%)	37 (23.6%)	
> High school diploma	1,381 (63.7%)	1,265 (63.3%)	116 (68.2%)	
**Marital status**				0.057
Married/Cohabitation	1,560 (65.6%)	1,453 (66.1%)	107 (60.3%)	
Widow/Divorced/Separated	356 (15.5%)	316 (14.6%)	40 (24.7%)	
Unmarried	484 (18.9%)	460 (19.3%)	24 (15.1%)	
**PIR**				0.333
≤1.3	697 (22.1%)	664 (22.1%)	33 (22.5%)	
1.3–3.5	833 (42.8%)	754 (42.1%)	79 (49.6%)	
>3.5	870 (35.1%)	811 (35.8%)	59 (27.9%)	
BMI (kg/m²)	26.2 (22.8, 32.2)	26.3 (22.9, 32.3)	25.5 (22.8, 30.6)	0.300
**Smoking**				0.321
Before smokers	399 (16.8%)	367 (16.5%)	32 (20.0%)	
Never smokers	1,480 (57.6%)	1,386 (58.3%)	94 (50.5%)	
Current smokers	521 (25.6%)	476 (25.3%)	45 (29.5%)	
**Alcohol drinking**				0.261
No	908 (31.5%)	856 (32.0%)	52 (26.2%)	
Yes	1,492 (68.5%)	1,373 (68.0%)	119 (73.8%)	
**Diabetes**				0.244
No	2,259 (94.4%)	2,095 (94.2%)	164 (96.6%)	
Yes	141 (5.6%)	134 (5.8%)	7 (3.4%)	
**Hypertension**				0.119
No	1,697 (67.8%)	1,601 (68.5%)	96 (60.4%)	
Yes	703 (32.2%)	628 (31.5%)	75 (39.6%)	
CHO (gm)	220.2 (167.5, 283.1)	220.6 (166.8, 281.6)	216.6 (173.8, 290.1)	0.512
Fiber (gm)	12.6 (9.0, 18.3)	12.7 (9.0, 18.4)	12.0 (9.0, 16.4)	0.256
CFR	17.2 (12.8, 23.0)	17.1 (12.6, 22.9)	17.8 (14.4, 23.8)	0.067
Albumin (g/dL)	4.2 (4.0, 4.4)	4.2 (4.0, 4.4)	4.2 (3.9, 4.4)	0.220
Segmented neutrophils (1,000 cell/uL)	4.4 (3.3, 5.6)	4.4 (3.3, 5.6)	4.3 (3.5, 5.3)	0.858
White blood cell count (1,000 cells/uL)	7.4 (6.1, 8.9)	7.4 (6.1, 9.0)	7.2 (5.9, 8.6)	0.522
NPAR	14.2 (12.7, 16.0)	14.2 (12.7, 16.0)	14.9 (13.3, 16.3)	0.072
Age at menarche	12 (12, 13)	12 (12, 13)	12 (12, 13)	0.996
**CFR (quartile)**				0.207
Q1 (<13.2)	600 (26.8%)	569 (27.7%)	31 (17.6%)	
Q2 (13.2–17.4)	600 (24.6%)	552 (24.2%)	48 (28.5%)	
Q3 (17.4–23.1)	600 (24.2%)	548 (23.8%)	52 (27.8%)	
Q4 (≥23.1)	600 (24.4%)	560 (24.3%)	40 (26.1%)	

**Notes:** Categorical variables are expressed in *n* (%), and continuous variables are expressed in mean (standard deviation SD]) or median (interquartile range IQR]); *n* is not weighted, while *n* (%), mean, SD and median IQR] are weighted. Within each CFR quartile, the unweighted sample size is 600 by design (equal-sized quartiles), while the accompanying percentages are survey-weighted, and thereby do not necessarily equal 25%. NHANES, the National Health and Nutrition Examination Survey; EM, endometriosis; PIR, poverty income ratio; BMI, body mass index; CHO, carbohydrate; CFR, carbohydrate-to-fiber ratio; NPAR, neutrophil percentage-to-albumin ratio.

### 3.2. Association of CFR with EM

**Figure 2.**
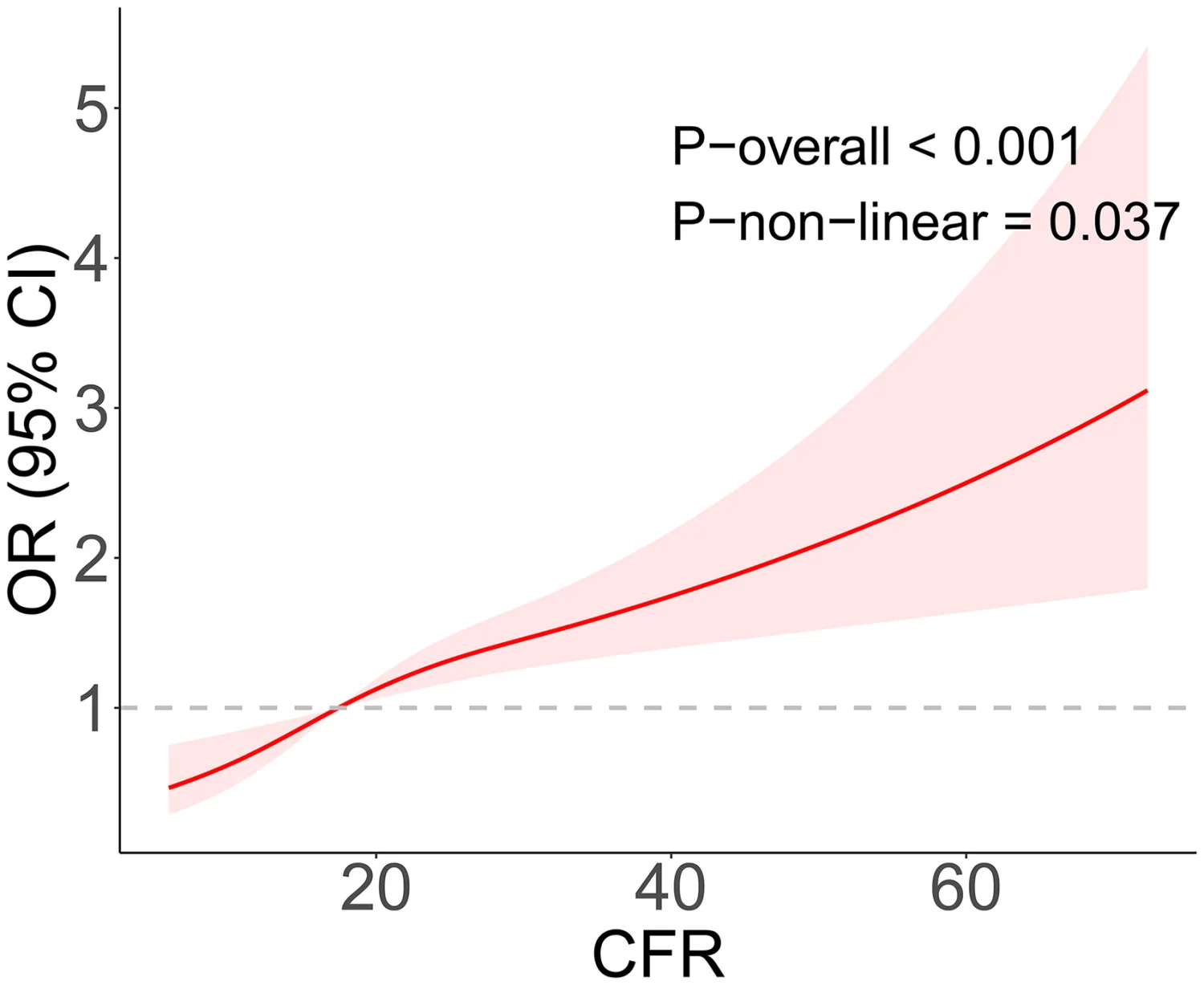
Association between the carbohydrate-to-fiber ratio (CFR) and endometriosis (EM). Adjusted for age, race, education, poverty income ratio (PIR), marital status, body mass index (BMI), smoking, alcohol drinking, diabetes, hypertension, and age at menarche.

As shown in Table 2, weighted logistic regression models were constructed to assess the association between CFR and EM. CFR was positively associated with EM across all models (crude: OR [95% CI]: 1.02 [1.00, 1.04]; model I: OR [95% CI]: 1.02 [1.01, 1.04]; model II: OR [95% CI]: 1.02 [1.01, 1.04]). After categorizing into quartiles, participants in Q2 (OR [95% CI]: 2.13 [1.01, 4.48]), Q3 (OR [95% CI]: 2.13 [1.01, 4.48]), and Q4 (OR [95% CI]: 2.17 [1.06, 4.41]) had higher odds of EM in the fully adjusted model, when compared with Q1. The fully adjusted odds ratios for Q2 and Q3 were essentially identical (both, OR = 2.13, 95% CI: 1.01–4.48). After full covariate adjustment and rounding to two decimals, the two middle quartiles converged on a similar elevated-odds plateau relative to Q1, consistent with the nonlinear, threshold-shaped association described below, in which the odds of EM rose steeply above the lowest quartile, and subsequently leveled off. The restricted cubic spline analysis revealed a nonlinear association between CFR and EM (*p* for nonlinearity = 0.037), with an inflection point at 17.3 (Figure 2). The piecewise linear regression revealed that when CFR was ≤17.3, each one-unit increase in CFR was associated with 11% higher odds of EM (OR [95% CI]: 1.11 [1.00, 1.22]), while no significant association was observed when CFR was >17.3 (OR [95% CI]: 1.02 [1.00, 1.04]) (Supplementary Table 1).

**Table 2.** Association of CFR with EM.

OR (95% CI)						
Participants	Crude	*p*-value	Model I	*p*-value	Model II	*p*-value
**CFR**						
Continuous	1.02 (1.00, 1.04)	0.020	1.02 (1.01, 1.04)	0.004	1.02 (1.01, 1.04)	0.009
**Classify**						
Q1 (<13.2)	ref.		ref.		ref.	
Q2 (13.2–17.4)	1.85 (1.01, 3.41)	0.047	2.14 (1.08, 4.24)	0.031	2.13 (1.01, 4.48)	0.047
Q3 (17.4–23.1)	1.84 (1.01, 3.34)	0.046	2.18 (1.07, 4.47)	0.034	2.13 (1.01, 4.48)	0.048
Q4 (≥23.1)	1.70 (0.94, 3.07)	0.079	2.25 (1.14, 4.44)	0.022	2.17 (1.06, 4.41)	0.036

Notes: Crude: non-adjusted. Model I: adjusted for age, race, education, poverty income ratio (PIR) and marital status. Model II: adjusted for age, race, education, PIR, marital status, body mass index (BMI), smoking, alcohol drinking, diabetes, hypertension, and age at menarche. CFR, carbohydrate-to-fiber ratio; EM, endometriosis; OR, odds ratio; CI, confidence interval.

### 3.3. Subgroup analysis

In order to further confirm the association between CFR and EM, subgroup analyses were performed. Positive associations were observed across multiple subgroups, which included the following: participants with a high school diploma or equivalent, participants who were married or cohabitating, participants who were never married, participants with PIR ≤1.3, never smokers, current smokers, participants who did not consume alcohol, participants without diabetes, and participants with or without hypertension. The interaction tests indicated no significant effect modification in any subgroup, except for hypertension (*p* for interaction = 0.040) (Supplementary Table 2).

### 3.4. Mediating role of NPAR in the association of CFR with EM

**Figure 3.**
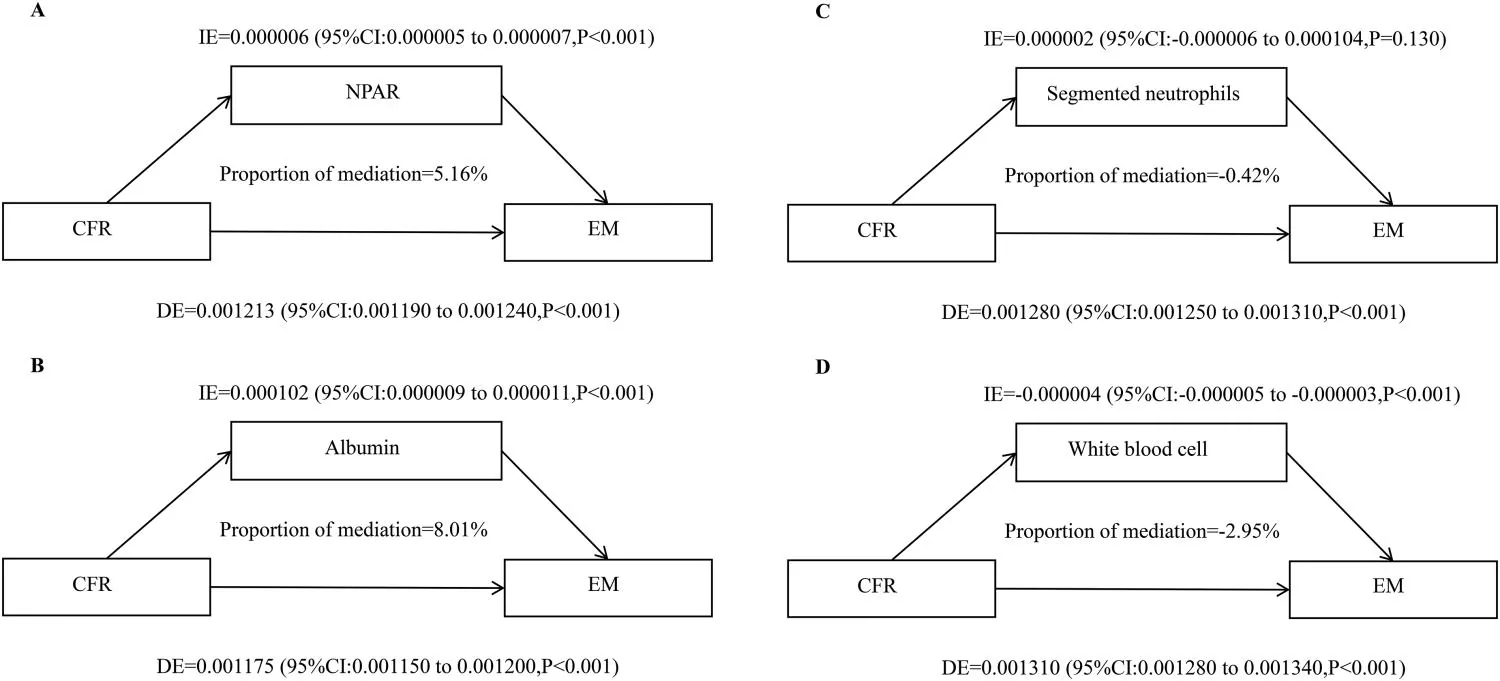
Mediation of the association between the carbohydrate-to-fiber ratio (CFR) and endometriosis (EM) through the neutrophil percentage-to-albumin ratio (NPAR) and its components. (A) NPAR; (B) albumin; (C) segmented neutrophils; (D) white blood cells. The detailed indirect-effect, direct-effect, confidence-interval, and mediation-proportion estimates are reported in the main text (Section 3.4). CFR, carbohydrate-to-fiber ratio; EM, endometriosis; NPAR, neutrophil percentage-to-albumin ratio; IE, indirect effect; DE, direct effect; CI, confidence interval.

A mediation analysis was performed to evaluate the potential mediating effect of NPAR on the association between CFR and EM. NPAR partially mediated this relationship, accounting for 5.16% of the total effect (indirect effect = 0.000006, 95% CI: 0.000005–0.000007; direct effect = 0.001213, 95% CI: 0.001190–0.001240; both, *p* < 0.001) (Figure 3(A)). The further analysis identified neutrophils, white blood cells, and albumin as potential mediators. As shown in Figure 3, albumin partially mediated the CFR-EM association (mediated proportion = 8.01%; indirect effect = 0.000102, direct effect = 0.001175, both *p* < 0.001), while white blood cells had a negative mediated proportion of −2.95% (indirect effect = −0.000004, direct effect = 0.001310, both *p* < 0.001), and segmented neutrophils had no significant mediating effect (mediated proportion = −0.42%, *p* = 0.130). When CFR was analyzed as a categorical variable, the mediation results for NPAR remained consistent (Supplementary Figure 1).

### 3.5. Sensitivity analysis

When unweighted data were used (Supplementary Table 3), the results were generally consistent with those of the main article, showing a significant positive association between CFR and EM. Subsequently, based on Model 3, the main analysis process was repeated while sequentially adjusting for dietary protein intake, energy intake, and physical activity. The conclusions remained consistent with the main findings of the article (Supplementary Table 4). Finally, after applying multiple imputation for missing covariates, a logistic regression model was constructed again to assess the association between CFR and EM. The conclusion remained consistent with the main results of the article (Supplementary Table 5).

## 4. Discussion

### 4.1. Principal findings

To our knowledge, the present population-based study was the first to evaluate the association between CFR and EM. The present study revealed a positive independent association between CFR and EM. After adjusting for sociodemographic, reproductive, and lifestyle characteristics, every 1-SD increment in CFR was associated with a 2% higher odds of EM (OR: 1.02, 95% CI: 1.01–1.04). Furthermore, a nonlinear association was found between CFR and EM, with a threshold value of 17.3. Participants with a CFR below 17.3 had an 11% increase in odds of EM for every 1-SD increment in CFR (OR: 1.11, 95% CI: 1.00–1.22), while no significant association was observed in participants with a CFR above the threshold value. Next, the mediation effect of NPAR on the association of CFR with EM was further investigated. The present results indicated that NPAR partially mediated the association between CFR and EM. The percentage of mediation was 5.16%. These findings may improve the understanding of the relationship between dietary quality and EM, and provide an opportunity to better monitor and stratify the risk of EM in the future.

The apparent contrast between the modest per-unit odds ratio in the continuous analysis (OR = 1.02) and the roughly two-fold odds ratios in the quartile analysis reflect two different reference frames, rather than conflicting results: the continuous estimate express the change in the odds of EM per one-unit increase in CFR, while the quartile estimates compare each higher quartile with the lowest (reference) quartile. Furthermore, since the continuous model assumes a linear association on the log-odds scale, it may underestimate an association that is concentrated at higher CFR levels. Categorizing the CFR into quartiles relaxes this assumption, and captures the threshold-shaped relationship identified by the restricted cubic spline.

### 4.2. Comparison with existing literature on dietary factors

Prior research that examined the association between specific dietary components and EM have reported mixed results, particularly with regard to carbohydrates and fiber [16,37]. The present study addressed these discrepancies by highlighting the significance of the relative proportions of carbohydrates and fiber in a diet. In previous case-control studies, high dietary fiber intake has been linked to reduced risk of EM, which is likely due to its anti-inflammatory effects and impact on estrogen metabolism [38,39]. However, prospective cohort studies have found no association, which may be attributed to the difference in dietary assessment, the lack of consideration for the quality of carbohydrates, or the potential inadequacy of models to account for the combined effects of pro-inflammatory and anti-inflammatory nutrients. Notably, the Nurses’ Health Study II observed no significant relationship between total carbohydrate intake and EM risk, when individually assessed, aligning with the present findings of comparable absolute carbohydrate intake in both the EM and control groups [40]. Nevertheless, the present study contributes a novel perspective by introducing the CFR concept, which encapsulates the complex interplay between refined carbohydrates and dietary fiber. Refined carbohydrates can drive inflammation by inducing sharp glycemic spikes, while fiber has multiple anti-inflammatory pathways. CFR emerged as a ratio that inherently reflects the combined influence of these two dietary components, recognizing that the pathogenic potential of high carbohydrate consumption may be contingent on a concomitantly low fiber intake. Conversely, a high fiber intake may mitigate the pro-inflammatory impact of carbohydrates [12]. The present research suggests that future studies in nutritional epidemiology might benefit from focusing on nutrient ratios and overall dietary patterns, rather than isolated nutrients, since these approaches are more representative of real-world dietary patterns and nutrient interactions.

The present findings both complement and extend recent evidence from prospective cohort studies. The NHSII reported that adherence to healthy dietary patterns (AHEI-2010) was inversely associated with EM diagnosis (HR for 5^th^ *vs.* 1^st^ quintile: 0.87, 95% CI: 0.78–0.96), while the Western dietary pattern exhibited positive associations (HR: 1.27, 95% CI: 1.09–1.47). These associations were particularly pronounced among participants without infertility, suggesting that dietary quality may influence EM risk through effects on pain presentation. The present CFR findings are consistent with these observations (OR: 2.17, 95% CI: 1.06–4.14 for highest *vs.* lowest quartile), since CFR captures the core elements that distinguish healthy from unhealthy eating patterns – refined carbohydrates characterize Western diets, while fiber characterizes healthy dietary patterns [37].

However, CFR offers distinct advantages as a clinically translatable metric. First, it is a simple, calculable ratio derived from standard dietary assessments without requiring complex scoring algorithms, making it highly accessible for clinical implementation. Second, CFR provides mechanistic focus on the specific carbohydrate-fiber interplay, as demonstrated by the present NPAR mediation analysis. Third, CFR translates directly into actionable recommendations – maintaining a ratio below 17 by reducing refined carbohydrates and increasing fiber intake. Fourth, the identification of a non-linear relationship with a threshold (CFR = 17.3) provides a concrete, feasible target for dietary intervention. Below this threshold, each unit increase in CFR was associated with 11% higher EM risk (OR: 1.11, 95% CI: 1.00–1.22), while above the threshold, the association was attenuated (OR: 1.02, 95% CI: 1.00–1.04).

These findings address the limitations of previous studies that separately examined carbohydrates and fiber, which yielded inconsistent results [38–40]. The NHSII observed no significant relationship between total carbohydrate intake and EM when individually assessed, aligning with the present findings of comparable absolute carbohydrate intake between groups [40]. Similarly, fiber studies have reported mixed results [41,42]. The inconsistencies underscore a key principle: the pathogenic potential of nutrients depends on overall dietary context [43]. High carbohydrate intake may only confer increased EM risk when accompanied by low fiber intake, while adequate fiber may mitigate the pro-inflammatory effects of carbohydrates [44]. This synergistic relationship cannot be captured by examining nutrients in isolation, highlighting the value of ratio-based approaches, such as the CFR, which more accurately represents real-world dietary patterns and nutrient interactions.

### 4.3. Biological mechanisms and inflammatory pathways

The significant correlation between higher CFR and greater risk of EM is consistent with the present understanding of inflammatory mechanisms in the disease. The consumption of a diet high in CFR likely indicates a pattern of eating, which involves refined carbohydrates and high glycemic load, as well as low intake of fiber. These nutritional factors are associated with systemic inflammation, which is central to the pathophysiology of EM [12]. The mechanisms through which this might occur include the following: frequent hyperglycemic spikes, the resultant insulin release that promotes oxidative stress and the production of advanced glycation end-products (AGEs), the activation of pro-inflammatory pathways, such as NF-κB, and the induction of a pro-inflammatory state [41]. Chronic hyperinsulinemia and hyperglycemia also lead to increased production of IGF-1 and decreased levels of sex hormone-binding globulin, resulting in higher circulating levels of estrogen, which has a role in the pathogenesis of EM [42]. On the other hand, high fiber intake induces anti-inflammatory effects through various pathways, including but not limited to the production of short-chain fatty acids (SCFAs), which have regulatory roles in the immune system and serve as an energy source for colonocytes [27]. Furthermore, fiber increases insulin sensitivity, promotes a favorable gut microbiota composition, and reduces estrogen levels, all of which may be beneficial in the context of EM [43]. NPAR was identified as a particularly informative metric in the present study, since it integrates both the inflammatory and nutritional aspects of the condition. A higher neutrophil percentage may reflect a state of chronic inflammation and immune activation, which are involved in the pathogenesis of EM. Neutrophils are involved in the formation of endometriotic lesions through the release of proteolytic enzymes, reactive oxygen species (ROS), and pro-angiogenic factors that support the survival of ectopic endometrial tissues. A lower albumin level may indicate poor nutritional status, increased inflammation (since inflammation inhibits albumin production through the liver), and oxidative stress [44]. The present mediation analysis revealed that NPAR partially mediates the relationship between CFR and EM, supporting the idea that diet may be associated with EM through inflammatory mechanisms. However, the present estimates suggest that this may not be the only or primary mechanism involved, since NPAR was found to mediate 5.16% of the effect. The other potential pathways through which dietary quality might affect EM include the following: direct hormonal effects, modulation of prostaglandin production, matrix metalloproteinase activity, and epigenetic modifications. It is noteworthy that the chronic inflammatory state induced by a poor diet can create a permissive environment for the implantation and survival of ectopic endometrial tissues, and its neovascularization and resistance to apoptosis, which are key processes in the development of EM.

### 4.4. Clinical and public health implications

The cut-off point of 17.3 was clinically significant, because this translates into an actionable and memorable dietary recommendation for the prevention of EM. In other words, based on the present findings, practical advice would be to keep the ratio below 17. This can be implemented by advising women to limit their consumption of refined carbohydrates, and eat more whole grains, legumes, fruits, and vegetables. This simple, cost-effective approach can be easily applied in clinical practice, as part of the risk assessment for EM, and as part of the dietary advice given to women. In turn, this can lead to a significant reduction in individual and social costs of EM. In addition, the dietary recommendations mentioned above are complementary to the medical and surgical treatment of EM. Most importantly, the proposed dietary recommendations will have wide public health implications, and can easily be used at the population level, in order to lower the risk of EM. It is noteworthy that the dietary advice on the intake of carbohydrates and fibers can even be more important for women with high blood pressure or women with low socioeconomic status (SES), since these individuals might have a greater risk of developing metabolic and inflammatory stress in response to a less healthy diet.

In practical terms, the CFR can be obtained directly from routinely available nutrition information by dividing the total daily carbohydrate intake (g) by the total daily fiber intake (g). For illustration, a breakfast of refined cereal with fruit juice may provide roughly 60 g of carbohydrates with only approximately 2 g of fiber, yielding a CFR of approximately 30 (well above the threshold), while a meal built on whole grains, legumes, vegetables, and fruit may provide roughly 45 g of carbohydrates with approximately 12 g of fiber, yielding a CFR of approximately 4 (well below the threshold). Patients and clinicians can thereby estimate the ratio using standard food labels or widely available nutrition-tracking mobile applications that report carbohydrate and fiber content. At present, no validated CFR-specific tool or application has been developed. Thus, creating and validating such a tool represents a useful direction for future work.

### 4.5. Study limitations

Although the present study benefits from the nationally representative NHANES data, rigorous quality control, advanced statistical modeling, and extensive covariate adjustments, the cross-sectional design of the study precludes causal inference, the reliance on self-reported EM may have introduced misclassification bias, the short-term dietary recalls limited the assessment of habitual intake, the residual and selection confounding could not be fully excluded, the mediation by NPAR only explained a small portion of the observed association, and the restriction to two NHANES cycles constrained the sample size and subgroup power. Thus, all of these warrant the cautious interpretation of these findings. In addition, although the indirect effect mediated by NPAR was statistically significant, it was very small in absolute terms (indirect effect ≈ 0.000006). Therefore, its clinical relevance is limited, and the mediation findings should be regarded as hypothesis-generating, rather than as evidence of a quantitatively important pathway.

## 5. Conclusion

In conclusion, CFR may be a useful dietary metric associated with EM prevalence that complements and extends previous research on dietary patterns. Although recent prospective studies have demonstrated associations between overall dietary quality and EM prevalence [37], the present study was the first to examine CFR as a specific, calculable ratio, and identify a concrete threshold value for risk stratification. CFR has the potential to be utilized as a simple, actionable metric in clinical and public health settings. Based on the present findings, maintaining a CFR below 17 appears to be a practical target for EM prevention, providing a cost-effective and easily accessible intervention strategy that can be readily communicated to patients, and implemented through dietary counseling focused on reducing refined carbohydrate intake, while increasing fiber consumption from whole grains, fruits, vegetables, and legumes.

## Supplementary Material

Supplementary.doc

## Data Availability

The data analyzed in the study are publicly available from the National Health and Nutrition Examination Survey (NHANES) website. The analytical code used in this study is available from the corresponding author upon reasonable request.

## Abbreviations

body mass index: BMI
carbohydrate-to-fiber ratio: CFR
Centers for Disease Control: CDC
Endometriosis: EM
National Health and Nutrition Examination Survey: NHANES
neutrophil percentage-to-albumin ratio: NPAR
odds ratio: OR
poverty income ratio: PIR
reactive oxygen species: ROS
standard deviation: SD
socioeconomic status: SES
