# Supplementary material for: Association between carbohydrate-to-fiber ratio and endometriosis in U.S. women: mediating role of neutrophil-percentage-to-albumin ratio
Source: Ann Med. 2026 Sep 19;58(1):2725512. doi: 10.1080/07853890.2026.2725512 (PMC13592008; doi:10.1080/07853890.2026.2725512)
Supplement: Supplementary file 1 [file IANN_A_2725512_SM7709.doc]

**Supplementary Figure 1 Estimated proportion for the association between endometriosis (EM) and the carbohydrate-to-fiber ratio (CFR) mediated by the neutrophil percentage-to-albumin ratio (NPAR).**

**Supplementary Table 1. Threshold effect analysis of CFR with EM by two-piecewise linear regression.**

|  | **Adjusted OR (95% CI)** | ***p*-value** |
| --- | --- | --- |
| **Inflection point** | 17.3 |  |
| CFR ≤17.3 | 1.11 (1.00, 1.22) | 0.024 |
| CFR >17.3 | 1.02 (1.00, 1.04) | 0.053 |
| **p for Log-likelihood ratio** | 0.004 |  |

**Notes:** Adjusted for age, race, education, poverty income ratio (PIR), marital status, body mass index (BMI), smoking, alcohol drinking, diabetes, hypertension, and age at menarche. CFR, carbohydrate-to-fiber ratio; EM, endometriosis; OR, odds ratio; CI, confidence interval

**Supplementary Table 2. Subgroup analysis for the association between CFR and EM.**

| **Participants** | **OR (95%CI)** | ***p*-value** | ***p* for interaction** |
| --- | --- | --- | --- |
| **Education** |  |  | 0.588 |
| <High school diploma | 1.00 (0.98, 1.03) | 0.700 |  |
| High school diploma/equivalent | 1.03 (1.01, 1.06) | 0.019 |  |
| >High school diploma | 1.03 (1.00, 1.05) | 0.035 |  |
| **Marital status** |  |  | 0.149 |
| Married/Cohabitation | 1.03 (1.01, 1.06) | 0.011 |  |
| Widow/Divorced/Separated | 1.00 (0.98, 1.03) | 0.700 |  |
| Unmarried | 1.08 (1.02, 1.13) | 0.009 |  |
| **PIR** |  |  | 0.240 |
| ≤1.3 | 1.05 (1.03, 1.07) | <0.001 |  |
| 1.3–3.5 | 0.99 (0.97, 1.02) | 0.500 |  |
| >3.5 | 1.02 (1.00, 1.05) | 0.076 |  |
| **Smoking** |  |  | 0.526 |
| Before smokers | 0.98 (0.91, 1.04) | 0.500 |  |
| Never smokers | 1.03 (1.00, 1.05) | 0.038 |  |
| Current smokers | 1.03 (1.00, 1.05) | 0.041 |  |
| **Alcohol drinking** |  |  | 0.312 |
| No | 1.04 (1.02, 1.06) | <0.001 |  |
| Yes | 1.01 (0.99, 1.04) | 0.200 |  |
| **Diabetes** |  |  | 0.056 |
| No | 1.02 (1.01, 1.04) | 0.008 |  |
| Yes | 0.84 (0.67, 1.06) | 0.120 |  |
| **Hypertension** |  |  | 0.040 |
| No | 1.02 (1.00, 1.03) | 0.047 |  |
| Yes | 1.04 (1.01, 1.06) | 0.004 |  |

**Notes:** Adjusted for age, race, education, poverty income ratio (PIR), marital status, body mass index (BMI), smoking, alcohol drinking, diabetes, hypertension, and age at menarche. CFR, carbohydrate-to-fiber ratio; EM, endometriosis; OR, odds ratio; CI, confidence interval; PIR, poverty income ratio

**Supplementary Table 3. Relationship between CFR and EM (Unweighted).**

| **Participants** | **Crude** |  | **Model I** |  | **Model II** |  |
| --- | --- | --- | --- | --- | --- | --- |
|  | **OR (95% CI)** | ***p*-value** | **OR (95% CI)** | ***p*-value** | **OR (95% CI)** | ***p*-value** |
| **CFR** |  |  |  |  |  |  |
| Continuous | 1.01 (1.00, 1.02) | 0.020 | 1.02 (1.01, 1.03) | 0.001 | 1.02 (1.01, 1.03) | 0.002 |
| **classify** |  |  |  |  |  |  |
| Q1 (<13.2) | ref. |  | ref. |  | ref. |  |
| Q2 (13.2-17.4) | 1.60 (1.01, 2.57) | 0.05 | 1.76 (1.09, 2.86) | 0.021 | 1.80 (1.12, 2.95) | 0.016 |
| Q3 (17.4-23.1) | 1.74 (1.11, 2.79) | 0.018 | 2.03 (1.26, 3.30) | 0.004 | 1.96 (1.22, 3.21) | 0.006 |
| Q4 (≥23.1) | 1.31 (0.81, 2.14) | 0.300 | 1.69 (1.01, 2.87) | 0.049 | 1.63 (0.96, 2.79) | 0.073 |

**Notes:** Crude: non-adjusted. Model I: adjusted for age, race, education, poverty income ratio (PIR), and marital status. Model II: adjusted for age, race, education, PIR, marital status, body mass index (BMI), smoking, alcohol drinking, diabetes, hypertension, and age at menarche. CFR, carbohydrate-to-fiber ratio; EM, endometriosis; OR, odds ratio; CI, confidence interval

**Supplementary Table 4. Relationship between CFR and EM.**

| **Participants** | **OR (95%CI)** | ***p*-value** |
| --- | --- | --- |
| **Model II** | 1.02 (1.01, 1.03) | 0.002 |
| **Model III** | 1.02 (1.00, 1.04) | 0.028 |
| **Model V** | 1.03 (1.01, 1.04) | 0.003 |

**Notes:** Model II: adjusted for age, race, education, poverty income ratio (PIR), marital status, body mass index (BMI), smoking, alcohol drinking, diabetes, hypertension, and age at menarche. Model III: +protein +energy. Model V: +physical activity. CFR, carbohydrate-to-fiber ratio; EM, endometriosis; OR, odds ratio; CI, confidence interval

**Supplementary Table 5. Relationship between CFR and EM (multiple imputation).**

| **Participants** | **Crude** |  | **Model I** |  | **Model II** |  |
| --- | --- | --- | --- | --- | --- | --- |
|  | **OR (95% CI)** | ***p*-value** | **OR (95% CI)** | ***p*-value** | **OR (95% CI)** | ***p*-value** |
| **CFR** |  |  |  |  |  |  |
| Continuous | 1.02 (1.00, 1.03) | 0.015 | 1.02 (1.01, 1.04) | 0.004 | 1.02 (1.01, 1.04) | 0.008 |
| **classify** |  |  |  |  |  |  |
| Q1 (<13.2) | ref. |  | ref. |  | ref. |  |
| Q2 (13.2–17.6) | 1.90 (1.05, 3.47) | 0.036 | 2.20 (1.12, 4.32) | 0.025 | 2.17 (1.05, 4.50) | 0.039 |
| Q3 (17.6–23.2) | 1.96 (1.11, 3.46) | 0.021 | 2.36 (1.19, 4.64) | 0.017 | 2.27 (1.11, 4.65) | 0.029 |
| Q4 (≥23.2) | 1.80 (1.01, 3.23) | 0.047 | 2.43 (1.24, 4.78) | 0.013 | 2.32 (1.14, 4.74) | 0.025 |

**Notes:** Crude: non-adjusted. Model I: adjusted for age, race, education, poverty income ratio (PIR), and marital status. Model II: adjusted for age, race, education, PIR, marital status, body mass index (BMI), smoking, alcohol drinking, diabetes, hypertension, and age at menarche. Quartile cut-offs in the multiple-imputation dataset differ slightly from the complete-case cut-offs, because these were re-derived after imputation. CFR, carbohydrate-to-fiber ratio; EM, endometriosis; OR, odds ratio; CI, confidence interval
